# Variation in Plasma Levels of Apixaban and Rivaroxaban in Clinical Routine Treatment of Venous Thromboembolism

**DOI:** 10.3390/life12050705

**Published:** 2022-05-08

**Authors:** Sara Reda, Eva Rudde, Jens Müller, Nasim Shahidi Hamedani, Johannes Oldenburg, Bernd Pötzsch, Heiko Rühl

**Affiliations:** Institute of Experimental Hematology and Transfusion Medicine, University Hospital Bonn, Venusberg-Campus 1, 53127 Bonn, Germany; sara.reda1@uk-koeln.de (S.R.); s4evrudd@uni-bonn.de (E.R.); jens.mueller@ukbonn.de (J.M.); nasim.shahidi_hamedani@ukbonn.de (N.S.H.); johannes.oldenburg@ukbonn.de (J.O.); bernd.poetzsch@ukbonn.de (B.P.)

**Keywords:** apixaban, rivaroxaban, venous thromboembolism, anti-Xa activity

## Abstract

Direct oral anticoagulants (DOACs) apixaban and rivaroxaban are broadly used in the management of venous thromboembolism (VTE). Although not routinely required, measurement of their plasma concentration is advised for an increasing number of indications. Due to the lack of therapeutic ranges, current guidelines recommend reporting DOAC plasma levels together with expected levels from previous pivotal studies. The aim of this study was to assess DOAC level variation in a large VTE patient population. Drug concentrations determined by measurement of the anti-Xa-activity using drug-specific calibrators in citrated plasma samples from patients on rivaroxaban (n = 1471) or apixaban (n = 725) were analyzed. Observed 5th–95th percentile ranges of apixaban peak/trough levels (63–299/13–114 ng/mL for 5 mg, 37–161/7–68 ng/mL for 2.5 mg twice daily) were similar to previously reported mass-spectrometry-based reference data, and 10th–90th percentile ranges of rivaroxaban peak/trough levels (98–367/8–55 ng/mL for 20 mg, 51–211/5–27 ng/mL for 10 mg once daily) were even narrower. Age and drug levels correlated weakly (r ≤ 0.330). Drug levels measured repeatedly in subgroups of patients showed a strong correlation (r ≥ 0.773). In conclusion, anti-Xa-activity-based measurement of apixaban and rivaroxaban yields reliable results. However, the paucity of levels off-range underlines the need for evidence-based thresholds to better assist clinical decision making.

## 1. Introduction

Venous thromboembolism (VTE) comprises the disease entities deep venous thrombosis (DVT), pulmonary embolism (PE), and the combination thereof [1]. It is the third most common vascular disease after myocardial infarction and ischemic stroke, with an annual incidence of 1–2 in 1000 [2,3]. The cornerstone of VTE treatment is anticoagulant therapy, which is increasingly conducted using direct oral anticoagulants (DOACs) [4]. DOACs have been found to be noninferior to vitamin K antagonists (VKA), the longstanding standard of care, in the management of VTE in large randomized trials [5]. Due to their more constant pharmacokinetic profile in comparison to VKA, fewer interactions with diet, and no requirement of routine laboratory monitoring, DOACs are recommended as the first choice of anticoagulant for treatment of VTE in international guidelines [6].

The DOACs apixaban and rivaroxaban are direct, reversible inhibitors of free and prothrombinase-bound activated coagulation factor X (FXa) [7,8]. For treatment or secondary prophylaxis of VTE, apixaban is administered twice daily (BD, bis in die) at a dose of 5 or 2.5 mg (except for the first week after an event) and reaches its peak concentration 3–4 h post dosing [9]. Rivaroxaban is usually administered once daily (OD) at a dose of 20 or 10 mg, while doses of 15 mg BD or OD are used in the first 21 days of treatment or in special patient populations, respectively [10]. Its peak plasma concentration is observed after 2 h, and post-dose intervals of 2–4 and 20–28 h have been used in previous pharmacokinetic studies to assess peak and trough concentrations after intake of rivaroxaban for treatment or prophylaxis of VTE [11,12].

While DOACs do not require routine laboratory monitoring, assessment of anticoagulant activity in patients on DOACs may be required under emergent and nonemergent circumstances, such as bleeding, forthcoming medical procedures, or patient factors interfering with pharmacokinetics [13,14]. As therapeutic ranges are not established, current guidelines recommend communication of DOAC plasma levels together with expected DOAC levels from previously published studies [14,15]. For apixaban, these data can be found, in lack of other sources, in the summary of product characteristics (SmPC) [9]. In addition to the SmPC of rivaroxaban [10], two pivotal studies [11,12] have reported drug levels in patients receiving rivaroxaban for treatment or prophylaxis of VTE and are commonly referred to in guidelines and other literature [13,15,16]. An overview of these data is shown in Table 1.

In addition to the expected ranges, 50 and 30 ng/mL are important threshold levels of DOACs in plasma, as exceeding values are considered high enough to warrant antidote administration in serious bleeding and before urgent interventions with high bleeding risk, respectively (14).

While the above-mentioned reference data have been obtained in pivotal clinical studies, there is limited real-world data available. The aim of this study was to assess the variation of drug levels in patients receiving DOACs for management of VTE in a clinical routine setting.

## 2. Materials and Methods

### 2.1. Identification and Inclusion of Patients

This retrospective cohort study was conducted at the Institute of Experimental Hematology and Transfusion Medicine, Bonn, Germany, in thrombosis patients who received DOACs and were referred to the outpatient clinic for coagulation disorders of our institution between 2013 and 2019. The patients were referred in the context of thrombophilia testing, and DOAC levels were routinely measured in the absence of suspected acute (breakthrough) thrombosis or active bleeding. Demographic and clinical data were extracted from medical records. Data on plasma levels of apixaban, rivaroxaban, and D-dimer were retrieved from the database of the laboratory information system.

**Figure 1:**
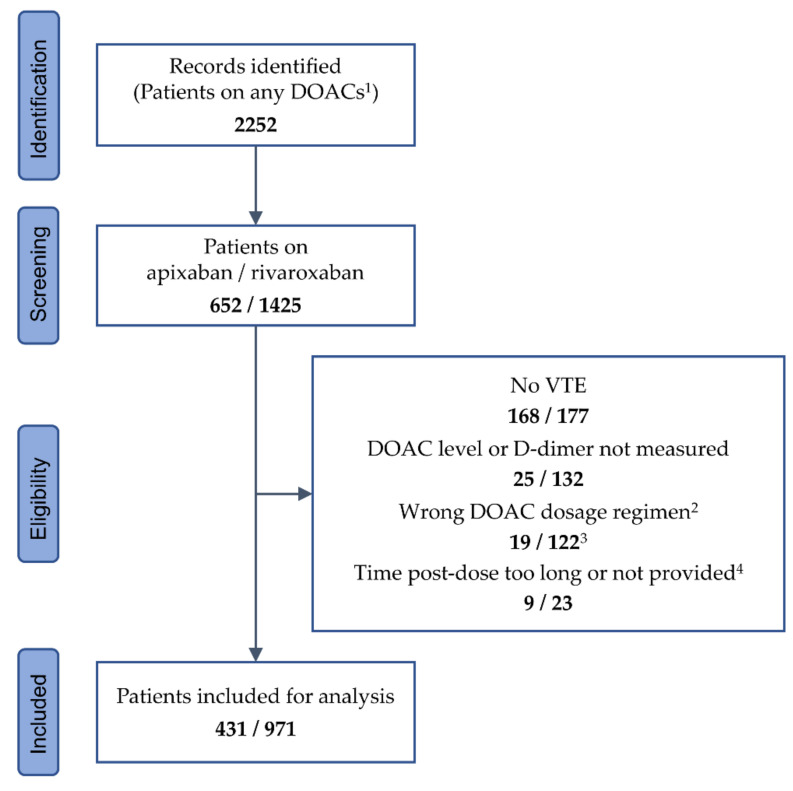
Identification and inclusion of patient data. ^1^ Including dabigatran (n = 92) and edoxaban (n = 83). ^2^ Any dosage regimen other than apixaban 5 or 2.5 mg twice daily, and rivaroxaban 20 or 10 mg once daily. ^3^ Thereof 43 patients taking rivaroxaban 15 mg once daily. ^4^ Time post-dose >18 h for apixaban, and >36 h for rivaroxaban. DOAC—direct oral anticoagulant; VTE—venous thromboembolism.

In the above-mentioned 7-year period, a total number of 2252 referred patients on DOACs were identified, thereof 652 on apixaban and 1425 on rivaroxaban, whose records were further screened. Patients were included if they received either apixaban 5 or 2.5 mg BD or rivaroxaban 20 or 10 mg OD. Patients receiving dabigatran or edoxaban were not included due to small cohort sizes. Included patients were required to have a history of VTE and measurements of plasma levels of DOACs and D-dimer performed at their visits. They were excluded if they received single or additional anticoagulant therapy with any drug or dosage other than listed above, if the time since the last intake of apixaban or rivaroxaban and/or the time of blood sampling were not documented, or if the post-dose interval exceeded 18 h in patients on apixaban and 36 h in patients on rivaroxaban. In total, 345 patients were excluded from the study population because they received apixaban or rivaroxaban for an indication other than VTE treatment, 157 patients because measurements of DOAC and/or D-dimer levels were not performed, 141 patients because of an anticoagulant dosage regimen not fulfilling the requirements, and 32 patients because the post-dose interval was too long or not provided. The remaining 1402 patients were included in the final analysis (Figure 1). Data from up to three follow-up visits were collected under the same eligibility conditions as for the first visit.

### 2.2. Measurement of Plasma Levels of DOACs and D-dimer

Blood samples were obtained from a venipuncture of a suitable arm vein using a 21-gauge winged infusion set (Sarstedt, Nümbrecht, Germany). After discarding the first 2 mL, blood was drawn into citrate tubes (10.5 mmol/L final concentration, Sarstedt). Plasma samples were obtained by centrifugation (2600× *g*, 10 min) within one hour after blood draw and assayed within 4 h. The Chromogenix Coamatic^®^ Heparin assay (Instrumentation Laboratory Company, Bedford, MA, USA) was used to determine the anti-Xa activity. Calibration curves based on rivaroxaban or apixaban (Technoview^®^, Technoclone, Vienna, Austria) were utilized to calculate the plasma concentrations of both drugs. D-dimer testing was performed using the Innovance^TM^ D-dimer assay, and all tests were performed on the BCS^®^ XP analyzer (Siemens Healthcare, Erlangen, Germany).

Preanalytics, anti-Xa activity, and D-dimer measurement in the laboratory of our institution were covered by accreditation with the national accreditation body and were performed according to ISO standards.

### 2.3. Statistical Analysis

The normality of data was tested using the Shapiro–Wilk test. Observed drug levels in plasma are presented as median and mean with percentile ranges or standard deviation (SD), respectively, regardless of the normality of data, in order to facilitate comparison with corresponding data from previous studies. A two-tailed Mann–Whitney test was performed to compare the patients’ age, weight, body mass index (BMI), and D-dimer level. The chi-square test was used for the comparison of frequencies. Spearman analysis was used to assess correlations. *p* values ≤ 0.05 were considered significant. All calculations were performed using the XLSTAT statistical and data analysis solution software (Addinsoft, Boston, MA, USA). All authors had access to primary data.

The study was conducted according to the guidelines of the Declaration of Helsinki and approved by the Institutional Review Board and Ethics Committee of the Medical Faculty of the University of Bonn (protocol code 236/05 and 070/05, approved on 10 June 2005).

## 3. Results

### 3.1. Study Population and Visits

Included over a 7-year period, the final study population consisted of 1402 patients (651 males and 751 females) with a history of VTE, thereof 431 patients on apixaban and 971 patients on rivaroxaban. The proportion of male and female patients differed between the cohorts (*p* = 4 × 10^−4^), as male patients were more frequent than female patients in the apixaban group, whereas in the rivaroxaban group, the ratio was balanced. At the first visit, D-dimer levels were higher in the apixaban cohort than in the rivaroxaban cohort, with 0.34 (0.22–0.53) mg/L in comparison to 0.27 (0.19–0.42) mg/L (*p* = 1.3 × 10^−7^), while age, body weight, and body mass index (BMI) did not show statistically significant differences between both groups (Table 2).

With 87/283 (20%) in comparison to 150/651 (15%), the frequency of patients with an isolated PE being their first event of VTE was slightly higher in the apixaban cohort than in the rivaroxaban cohort (*p* = 0.034), whereas the frequencies of DVT, PE, and of overall first and recurrent events of VTE did not differ significantly. In the rivaroxaban cohort, the proportion of first visits on the total number of visits included in the analysis was higher than in the apixaban cohort (*p* < 10^−4^), with 624/1471 (64%) in comparison to 237/725 (55%), while the proportion of consecutive visits on the total number of visits did not differ (Table 2). A total of 17% of the patients in the apixaban cohort and 14% of the patients in the rivaroxaban cohort received different dosages of the respective drugs during the study period.

### 3.2. DOAC Levels in the Study Population

**Figure 2:**
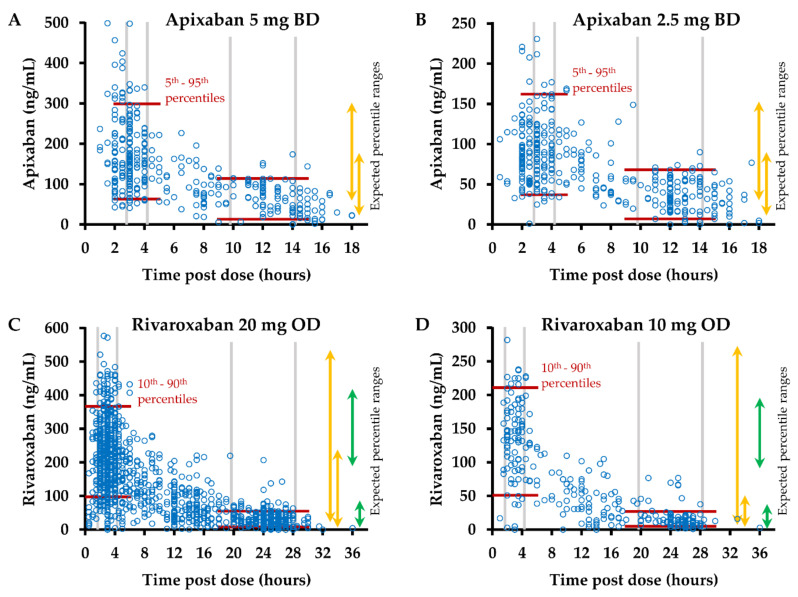
Plasma levels of apixaban and rivaroxaban. Anti-Xa activity was measured in plasma samples obtained at the indicated intervals following intake of (**A**) apixaban 5 mg twice daily (bis in die, BD) (n = 360), (**B**) apixaban 2.5 mg BD (n = 365), (**C**) rivaroxaban 20 mg once daily (OD) (n = 1180), and (**D**) rivaroxaban 10 mg OD (n = 291). Plasma concentrations were calculated using drug-specific calibrators. Red lines indicate the percentile ranges of observed plasma levels at peak and trough post-dose intervals. Arrows indicate the expected percentile ranges, 5th–95th percentile for apixaban (summary of product characteristics, SmPC [9]), 10th–90th percentile (orange, SmPC [10]) or 5th–95th percentile (green, Mueck et al. [11,12]) for rivaroxaban.

Plasma levels of apixaban and rivaroxaban in all samples are shown in Figure 2.

In total, 212 (29%) of all samples from patients on apixaban were obtained 3–4 h post-dose at the expected peak plasma concentration [9], and 173 (24%) samples were collected 10–14 h after the last intake of apixaban. Among the samples from patients on rivaroxaban, 556 (38%) and 416 (28%) were collected at the expected peak (2–4 h post-dose) and trough (20–28 h post-dose) plasma concentrations [10,11,12].

The observed median/mean values and percentile ranges in samples obtained at the expected peak and trough plasma concentrations of apixaban and rivaroxaban are listed in Table 3. For apixaban, observed 5th–95th percentile ranges were similar at peak and slightly lower at trough post-dose intervals in comparison to the ranges stated in the SmPC [9] (Figure 2A,B and Table 3). Observed 10th–90th percentile ranges for rivaroxaban were narrower than the ranges stated in the SmPC [10] but markedly wider than the 5th–95th percentile ranges reported by Mueck et al. [11,12] at peak post-dose intervals (Figure 2C,D and Table 3).

The highest variation in drug levels was observed in the rivaroxaban cohort at trough post-dose intervals, with coefficients of variation (CV) of 95% and 78% for rivaroxaban 10 mg OD and 20 mg OD, respectively. The CV ranged between 43% and 53% in all other subgroups of samples (Table 3).

The frequencies of samples with drug levels out of the expected ranges, based on the SmPCs [9,10], ranged between 5.0% and 13% throughout the subgroups. Drug levels out of these expected ranges were observed more frequently at peak post-dose intervals in the apixaban cohort (21/212, 10.0%) than in the rivaroxaban cohort (29/556, 5.2%, *p* = 0.029). At through post-dose intervals, the respective frequencies did not differ significantly. Based on the ranges reported by Mueck et al. [11,12], rivaroxaban levels out of the expected ranges were observed significantly more often at peak (233/556, 41.9%, *p* < 10^−4^) but not at trough post-dose intervals (identical frequencies of 32/416, 7.7%) (Table 3). Four patients on rivaroxaban 20 mg OD, aged between 63 and 84 years, had trough levels between 87 ng/mL [11] and 239 ng/mL [10]. None of the patients on apixaban or rivaroxaban 10 mg OD had trough levels above the expected range and was older than 60 years. Among the patients with trough levels below the expected range was only one with a BMI above 40 kg/m^2^, a female patient on rivaroxaban 10 mg OD, BMI of 50.2 kg/m^2^, with a drug level of 2 ng/mL measured 26 h post dose. The BMI of all other patients with trough levels below the expected range ranged between 18.4 and 34.8 kg/m^2^.

At peak post-dose intervals, the frequencies of samples with drug levels of no more than 50 or 30 ng/mL ranged from 0.8–15.2% and 0–5.0%, respectively, in the cohorts of the study. At trough post-dose intervals, drug levels of at least 50 or 30 ng/mL were observed in 4.8–63% and 8.7–83% of the samples, with the highest frequencies observed in patients on apixaban 5 mg BD (Table 3).

### 3.3. Dependence of DOAC Levels on Patient Characteristics

**Figure 3:**
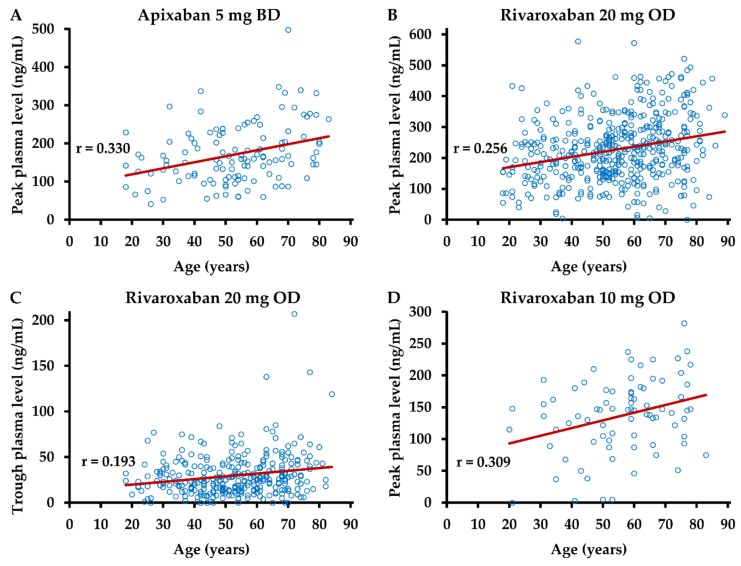
Dependence of drug levels on age. Anti-Xa activity was measured in plasma samples obtained (**A**) 3–4 h after intake of apixaban 5 mg (n = 72), (**B**) 2–4 h after intake of rivaroxaban 20 mg (n = 476), (**C**) 20–28 h after intake of rivaroxaban 20 mg (n = 312), and (**D**) 2–4 h after intake of rivaroxaban 10 mg (n = 80). Plasma concentrations were calculated using drug-specific calibrators. BD—bis in die, twice daily; OD—once daily; r—Pearson’s correlation coefficient.

In order to study potential associations between patient characteristics and observed plasma levels of DOACs more comprehensively, the correlation between age, body weight, BMI, and drug levels at peak and trough post-dose intervals was analyzed. A weak, albeit statistically significant (*p* ≤ 0.05), correlation between age and drug levels was observed in samples from patients on apixaban 5 mg BD or on rivaroxaban at peak and in samples from patients on rivaroxaban 20 mg OD at trough post-dose intervals (Figure 3).

**Figure 4:**
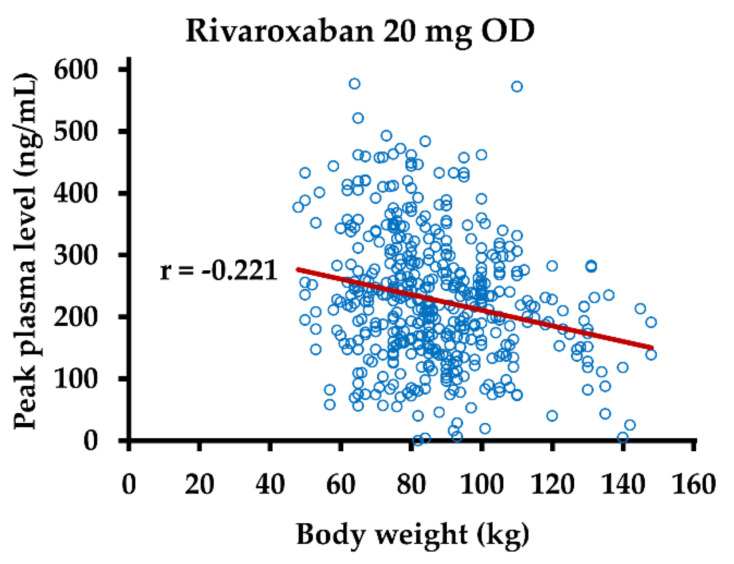
Dependence of rivaroxaban plasma levels on body weight. Anti-Xa activity was measured in plasma samples obtained 2–4 h after intake of rivaroxaban 20 mg (n = 476). Rivaroxaban plasma concentrations were calculated using drug-specific calibrators. OD—once daily; r—Pearson’s correlation coefficient.

In samples from patients on rivaroxaban 20 mg OD, a weak negative correlation between body weight and drug level at the peak post-dose interval was observed (Figure 4). 

There was no statistically significant correlation between age or body weight and DOAC levels in the other subgroups of samples and no correlation between BMI and drug levels in any subgroup. We assessed a potential association between plasma levels of DOACs and D-dimer in the same manner but did not find a statistically significant correlation in any subgroup. 

### 3.4. DOAC Levels Show Good Intra-Individual Agreement in Repeated Measurements

**Figure 5:**
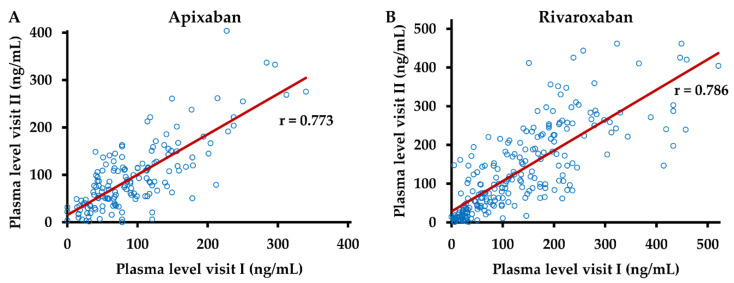
Intra-individual agreement of drug levels. Anti-Xa activity was measured in plasma samples obtained at two different visits (I and II) from patients following intake of (**A**) apixaban (n = 156) and (**B**) rivaroxaban (n = 234) at identical dosages and a difference of 4 h or less between post-dose intervals. Plasma concentrations of apixaban and rivaroxaban were calculated using drug-specific calibrators. r—Pearson’s correlation coefficient.

In order to assess the intra-individual variation of DOAC levels, we analyzed the correlation between plasma levels in samples, which had been obtained at two subsequent visits at identical dosages of apixaban and rivaroxaban and differences of four hours or less between the respective post-dose intervals (Figure 5).

For both DOACs, a strong positive correlation between these measurements was observed, with r = 0.773 for apixaban and r = 0.786 for rivaroxaban (*p* < 10^−4^ each).

### 3.5. Follow-Up of Patients Who Switched Anticoagulants

In 47 patients, anticoagulant treatment was switched between rivaroxaban and apixaban, and follow-up data on DOAC levels at peak or trough post-dose intervals were available. In total, 46 of these patients switched from rivaroxaban to apixaban, thereof 30 from rivaroxaban 20 mg OD to apixaban 2.5 mg BD, 10 to apixaban 5 mg BD, and 6 from rivaroxaban 10 mg OD to apixaban 2.5 mg BD. Rivaroxaban levels before this switch lay within the 10th–90th percentile in 28 (61%) patients; they were lower in 12 (26%) and higher in 6 (13%) patients, respectively. After the switch, apixaban levels lay within the 5th–95th percentile in 42 (91%) patients. They were lower in three (7%) patients and higher in one (2%) patient, three of them with rivaroxaban levels in the expected range before the switch. Thus, apixaban levels were adequate in 17 out of 18 patients (94%) who had too high or too low rivaroxaban levels before. One patient, who switched from apixaban 2.5 mg BD to rivaroxaban 20 mg OD due to a superficial venous thrombosis during treatment, had DOAC levels within the expected range before and after the switch.

## 4. Discussion

To our knowledge, this is the first study that reports dosage-specific apixaban and rivaroxaban plasma levels in a real-world VTE patient population large enough to allow comparison with reference data obtained in previous clinical studies. At trough post-dose intervals, drug level ranges observed in our study population were similar to those reported in the respective SmPCs [9,10] or by Mueck et al. [11,12], except for the cohort on rivaroxaban 20 mg OD, in which a markedly narrower 10th–90th percentile range was observed in comparison to the one stated in the SmPC [10]. However, in this cohort, as well as among all other groups, the frequency of observed drug levels outside the expected intervals (6.4–11.5%) lay in a typical range for 5th–95th and 10th–90th percentile intervals. There were some differences in the patient characteristics between the cohorts of apixaban and rivaroxaban users, such as sex distribution, frequency of patients with isolated PE, and proportion of patients with only one visit completed, but the frequency of drug levels outside the expected range did not differ significantly between cohorts. At peak post-dose intervals, observed drug level ranges were similar to those stated in the SmPC [9] only for patients on apixaban, whereas in the rivaroxaban cohort, they were narrower than the expected ranges stated in the SmPC [10] and wider than the ones stated by Mueck et al. [11,12]. The difference between the latter data and observed peak plasma levels of rivaroxaban can further be seen in the high frequency of measurements out of this expected range of 43.5% for rivaroxaban 20 mg OD and 32.5% for rivaroxaban 10 mg OD. These data suggest that the 5th–95th percentile ranges reported by Mueck et al. [11,12] are not well suited to serve as reference data at peak post-dose intervals. Overall, the good agreement of observed apixaban and rivaroxaban levels with expected plasma levels at trough post-dose intervals demonstrates the validity of both our data and of the expected ranges stated in the literature. This also applies to the observed and expected apixaban levels at peak post-dose intervals. In addition, it can also be interpreted as an indicator of the validity of the peak rivaroxaban levels observed in our study population that their 10th–90th percentile intervals ranged between those stated in the SmPC [10] and those reported by Mueck et al. [11,12]. It has been suggested that, if nonemergent testing is necessary, drug levels should be assessed at trough post-dose intervals, and our data support this recommendation [15].

Due to its high specificity, sensitivity, and reproducibility, mass spectrometry is considered the gold standard method for the measurement of DOACs [13] and has been used in the clinical development of apixaban and rivaroxaban to measure their plasma concentration and evaluate their pharmacokinetics [17,18,19]. Chromogenic anti-Xa activity assays are more widely used in clinical routine laboratories and have been demonstrated to be comparable to mass spectrometry in quantifying apixaban and rivaroxaban when combined with drug-specific calibrators [20,21,22], as was done in this study. The good agreement of anti-Xa activity-based measurement of drug levels in our study and previously reported mass spectrometry-based data [9,11,12] further strengthens the recommendation to use anti-Xa assays for the quantification of apixaban and rivaroxaban [15]. The suitability of these assays for the measurement of apixaban and rivaroxaban plasma levels in clinical routine is also supported by the strong correlation between subsequent measurements in the same patients in our study. Our data support a strategy of switching to apixaban if rivaroxaban plasma levels out of the expected range are observed, as apixaban levels were adequate in 17 out of 18 patients after this switch was performed.

The variation of apixaban or rivaroxaban plasma levels in real-world populations has been studied before in patients with nonvalvular atrial fibrillation (NVAF) [23,24,25,26] or in populations receiving these DOACs for different indications, including management of VTE [27,28,29]. Consistent with our results in VTE patients, Testa et al. [23] found an inter-patient CV of up to 42% at peak and up to 68% at trough levels of apixaban 5 or 2.5 mg BD and of up to 49% at peak and 103% at trough levels of rivaroxaban 20 mg OD in patients with NVAF. The dosage of rivaroxaban 10 mg OD was not administered in this indication. Wright et al. [27] and Bavalia et al. [28] included patients who received DOACs for the management of VTE but did not analyze drug-specific and/or dosage-specific variation of drug levels due to small sample sizes. In the study by Rottenstreich et al. [29], plasma levels out of the expected range were observed in 36/147 (24.5%) patients on apixaban and 33/102 (32.4%) patients on rivaroxaban. However, in this previous study, 82.5% of DOAC measurements were performed in special situations, including bleeding, perioperative assessment, or suspected breakthrough thrombosis, and only 17.5% were routine measurements [29], which might explain the higher rate of drug levels out of the expected range in comparison to our study.

Measurement of plasma DOAC concentrations has been recommended, amongst others, in patients with advanced age or a BMI above 40 kg/m^2^, and the DOAC plasma concentration should be in the range of concentrations observed in other populations [14]. Advanced age is associated with the risk of overdosing due to impaired elimination, whereas severe obesity bears the risk of underdosing. In our study, a weak positive correlation between age and plasma levels of apixaban and rivaroxaban was observed. While BMI and drug levels did not correlate negatively, there was a weak negative correlation between body weight and rivaroxaban plasma level, albeit only for peak levels and in the dosage group of 20 mg OD. Depending on the choice of reference data, four patients/no patient with rivaroxaban levels above the expected range aged over 60 years, and only a single patient with a BMI above 40 kg/m^2^ was among those with rivaroxaban levels below the expected range. This small number of patients with drug levels out of the expected range supports the established concept that routine monitoring of DOACs is not required. The observation that advanced age and severe obesity were identified as an explanation for DOAC levels out of the expected range in only a few patients and the overall very weak correlation of these variables to measured DOAC levels could suggest that other factors might have a greater influence on the variation of DOAC levels. DOAC levels of less than 50 or 30 ng/mL were observed in some patients at peak post-dose intervals, which emphasizes that decisions regarding antidote use should consider not only measurement results of DOAC levels but also the reported time of DOAC intake. In our study, the majority of patients on rivaroxaban 20 mg OD had trough levels of more than 30 or 50 ng/mL, supporting recommendations to withhold rivaroxaban prior to surgery for at least 24 h only when the procedural bleeding risk is low, but at least 48 h in case of uncertain, intermediate, or high risk of bleeding [30,31].

Due to the retrospective design of our study, DOACs were prescribed at the discretion of the treating physician, resulting in a real-world study population with an uneven distribution of patients on apixaban or rivaroxaban at different dosages. Factors not known to us might have influenced the selection of anticoagulant drugs and dosages by the treating physician, including hepatic or renal function, treatment adherence (e.g., intake of rivaroxaban 20 mg with food), concurrent medication, bleeding or thrombotic complications under ongoing or preceding anticoagulant treatment. These data were only sporadically documented in the medical records, which precluded statistical assessment. We did not analyze a potential relationship between DOAC levels and clinical outcomes. However, D-dimer levels in plasma were measured as a surrogate parameter of thrombotic risk but did not correlate with observed DOAC levels. We refrained from analyzing various patient characteristics of interest that are known to affect the plasma concentration of DOACs including, e.g., comorbidities such as renal or hepatic impairment, and comedication such as P-glycoprotein/cytochrome P3A4 inhibitors or inducers, as these data were available only sporadically, owing to the retrospective nature of the study. Further limitations of our study are that it is a single-center study and samples were taken opportunistically.

From a wider perspective, growing knowledge of the variation of DOAC levels and the availability of tests for their quantification in routine laboratories have led to the recommendation to measure DOAC levels in special patient populations and situations. However, there is still the need to define therapeutic ranges for the different DOACs to allow evidence-based clinical decisions from the results of DOAC monitoring in special patients. One might speculate if the availability of such therapeutic ranges will lead to a general paradigm shift in DOAC use towards monitoring of DOAC levels in broader patient populations.

## 5. Conclusions

The observed real-world variation of apixaban and rivaroxaban levels showed good agreement with reference data from clinical studies at trough post-dose intervals. The obtained data support recommendations to not measure DOAC levels routinely and to prefer measurement at trough over peak post-dose intervals in nonemergent situations. Further studies are warranted to determine clinically relevant thresholds of DOAC levels.

## Figures and Tables

**Table 1:** Expected drug levels in patients receiving DOACs for treatment or prophylaxis of VTE.

Drug and Dosage	Interval Post Dose (Hours)	Drug Level *, ng/mL	Reference
Apixaban 5 mg BD	Peak (3–4)	132 (59–302)	SmPC [9]
	Trough (about 12)	63 (22–177)	SmPC [9]
Apixaban 2.5 mg BD	Peak (3–4)	67 (30–153)	SmPC [9]
	Trough (about 12)	32 (11–90)	SmPC [9]
Rivaroxaban 20 mg OD	Peak (2–4)	270 (189–419)	Mueck et al. [11]
	Trough (20–28)	26 (6–87)	Mueck et al. [11]
	Peak (2–4)	215 (22–535)	SmPC [10]
	Trough (about 24)	32 (6–239)	SmPC [10]
Rivaroxaban 10 mg OD	Peak (2–4)	125 (91–196)	Mueck et al. [12]
	Trough (20–28)	9 (1–38)	Mueck et al. [12]
	Peak (2–4)	101 (7–273)	SmPC [10]
	Trough (about 24)	14 (4–51)	SmPC [10]

* Median (5th–95th percentile) for apixaban [9], mean (5th–95th percentile) [11] or (10th–90th percentile) [10] for rivaroxaban 20 mg OD, and median (5th–95th percentile) [12] or mean (10th–90th percentile) [10] for rivaroxaban 10 mg BD. BD (bis in die)—twice daily; DOAC—direct oral anticoagulant; OD—once daily; SmPC—summary of product characteristics; VTE—venous thromboembolism.

**Table 2:** Characteristics of the study population.

Drug	Apixaban	Rivaroxaban	*p* ^1^
Patients, n	431	971	-
Sex, male/female, n	169/262	482/489	4 × 10^−4^
Age, years (median, IQR) ^2^	55 (42–67)	53 (41–64)	ns
Body weight, kg (median, IQR) ^2^	83 (70–95)	83 (73–96)	ns
BMI, kg/m^2^ (median, IQR) ^2^	27.1 (24.3–30.8)	27.3 (24.4–30.8)	ns
D-dimer level, mg/L (median, IQR) ^2^	0.34 (0.22–0.53)	0.27 (0.19–0.42)	1.3 × 10^−7^
Patients with the first event of VTE, n (%)	283 (66%)	651 (67%)	ns
Thereof DVT, n (%)	121 (28%)	304 (31%)	ns
DVT with pulmonary embolism, n (%)	75 (17%)	197 (20%)	ns
Isolated pulmonary embolism, n (%)	87 (20%)	150 (15%)	0.034
Patients with recurrent VTE, n (%)	148 (34%)	320 (33%)	ns
Patients with one visit completed, n (%)	237 (55%)	626 (64%)	<10^−4^
Two visits completed, n (%)	121 (28%)	230 (24%)	ns
Three visits completed, n (%)	46 (11%)	75 (8%)	ns
Four visits completed, n (%)	27 (6%)	40 (4%)	ns
Visits included in the analysis, n	725	1471	-
Thereof on apixaban 5/2.5 mg twice daily, n (%)	360 (50%)/365 (50%)	-	-
Rivaroxaban 20/10 mg once daily, n (%)	-	1180 (80%)/291 (20%)	-

^1^ Mann–Whitney test for age, body weight, body mass index (BMI), and D-dimer level, chi-square test for frequencies. ^2^ At first visit. DVT—deep venous thrombosis; IQR—interquartile range; ns—not significant (>0.05); VTE—venous thromboembolism.

**Table 3:** Observed drug concentrations in plasma in relation to expected and critical levels.

	Drug	Apixaban		Rivaroxaban	
		5 mg BD	2.5 mg BD	20 mg OD	10 mg OD
Peak	Interval post dose, hours	3–4	3–4	2–4	2–4
	Samples, n	120	92	476	80
	Drug level, ng/Ml ^1^	160 (63–299)	90 (37–161)	227 (98–367)	137 (51–211)
	Mean ± SD, ng/mL (CV)	171 ± 78 (45%)	94 ± 41 (43%)	227 ± 104 (46%)	137 ± 59 (43%)
	Not in range [9,10], n (%)	9 (7.5%)	12 (13.0%)	24 (5.0%)	5 (6.4%)
	Not in range [11,12], n (%)	-	-	207 (43.5%)	26 (32.5%)
	≤50 ng/mL, n (%)	1 (0.8%)	14 (15.2%)	12 (2.5%)	8 (10.0%)
	≤30 ng/mL, n (%)	0	3 (3.3%)	8 (1.7%)	4 (5.0%)
Trough	Interval post dose, hours	10–14	10–14	20–28	20–28
	Samples, n	72	101	312	104
	Drug level, ng/mL ^1^	71 (13–114)	34 (7–68)	30 (8–55)	16 (5–27)
	Mean ± SD, ng/mL (CV)	70 ± 38 (53%)	37 ± 20 (46%)	30 ± 23 (78%)	16 ± 15 (95%)
	Not in range [9,10], n (%)	5 (6.9%)	7 (6.9%)	20 (6.4%)	12 (11.5%)
	Not in range [11,12], n (%)	-	-	24 (7.7%)	8 (7.7%)
	>50 ng/mL, n (%)	45 (63%)	32 (32%)	43 (13.8%)	5 (4.8%)
	>30 ng/mL, n (%)	60 (83%)	61 (60%)	189 (60.6%)	9 (8.7%)

^1^ Median (5th–95th percentile) for apixaban, mean (10th–90th percentile) for rivaroxaban. BD (bis in die)—twice daily; OD—once daily; SD—standard deviation; CV—coefficient of variation (SD/mean).

## Data Availability

The data presented in this study are available from the corresponding author.
